# Bone healing after low-level laser application in extraction sockets grafted with allograft material and covered with a resorbable collagen dressing: a pilot histological evaluation

**DOI:** 10.1186/s12903-015-0122-7

**Published:** 2015-10-29

**Authors:** Adriana Monea, Gabriela Beresescu, Mezei Tibor, Sorin Popsor, Dragos Mihai Antonescu

**Affiliations:** Department of Odontology and Oral Pathology, Faculty of Dental Medicine, University of Medicine and Pharmacy Tirgu-Mures, Tirgu Mureș, Romania; Department of Tooth Morphology and Dental Materials, Faculty of Dental Medicine, University of Medicine and Pharmacy Tirgu-Mures, Tirgu Mureș, Romania; Department of Morphopatology, Faculty of Medicine, University of Medicine and Pharmacy Tirgu-Mures, Tirgu Mureș, Romania; Department of Prosthetics and Oral Rehabilitation, Faculty of Dental Medicine, University of Medicine and Pharmacy Tirgu-Mures, Tirgu Mureș, Romania

**Keywords:** Low level laser therapy, Bone regeneration, Socket graft

## Abstract

**Background:**

Our aim was to determine whether low level laser therapy (LLLT) can decrease the time between extraction/socket graft and implant placement, by evaluating histological changes in sockets grafted with a particulate allograft material and treated with LLLT.

**Methods:**

Thirty patients had a socket grafted with a particulate allograft material (MinerOss) covered with a resorbable collagen wound dressing. The patients were then randomly divided into two equal groups (*n* = 15): test group receiving postoperative LLLT treatment, and control group without postoperative laser treatment. The assessment of bone formation was carried out in both groups at well-determined time intervals after surgery by histostomorphometric analysis.

**Results:**

The histological results of the site treated with LLLT for 21 days, harvested at 60 days after grafting showed abundant new bone formation without any sign of inflammation. The same results were obtained in the control group not before 120 days post-surgery.

**Conclusions:**

It can be concluded that LLLT photobiomodulation can reduce the healing time after grafting the extraction socket. Histological evidence suggests that new bone formation in the sockets appeared within 60 days after LLLT treatment compared to a minimum of 120 days in the control group.

## Background

Low level laser treatment (LLLT) has increased in popularity and is more frequently used as an adjuvant in the treatment in a various conditions in dentistry.

The process of bone regeneration, which includes proliferation and differentiation of the osteoblasts, matrix formation and calcification, is influenced by a series of factors - biomechanical, biochemical, cellular, hormonal and pathological [1]. It has been argued that LLLT may be supportive in the healing process by influencing various tissue responses such as blood flow, inflammation, cellular proliferation and cellular differentiation [1].

At low doses, LLLT has been shown to enhance cell proliferation in vitro in several types of cells: fibroblasts [1, 2], keratinocytes [3], endothelial cells [4], osteoblasts [5], lymphocytes [6, 7]. LLLT stimulate lymphocytes, activate mast cells and proliferation of various cell types therefore acting as anti-inflammatory [7]. Stein and collaborators showed that LLLT (He-Ne laser irradiation) promotes proliferation and maturation of humans osteoblasts in vitro [5].

The successful placement and integration of the dental implants in the previously grafted extraction sockets require adequate time for the healing and sufficient regeneration of the bone. A number of different studies showed that the healing time of an extraction socket grafted with a particulate allograft material can range from 4 to 6 months depending on the site of the defect [8, 9, 11]. A decrease in the time interval between the extraction/grafting time and the implant placement would be very beneficial to the patients. Experimental research has shown different methods to enhance bone regeneration such as mechanical stimulation [10, 11], low intensity ultrasound [12, 13], biological growth factors [14] and low level laser therapy [15].

The aim of this study was to determine whether LLLT can decrease the time between extraction/socket graft and implant placement, by evaluating histological changes in sockets grafted with a particulate allograft material and treated with LLLT.

Trial registration: ACTRN12615001013550.

## Methods

Thirty-five patients were included in the our study. Inclusion criteria were as follows: age over 20, non-smoker, systemically healthy, no chronic treatment for any systemic disease, no active infection present at the time of extraction. The study protocol had been approved by Ethical Committee of University of Medicine and Pharmacy Tirgu Mures, Romania (No 16/29.05.2014). All the patients recruited for the study signed an informed consent.

**Fig. 1:**
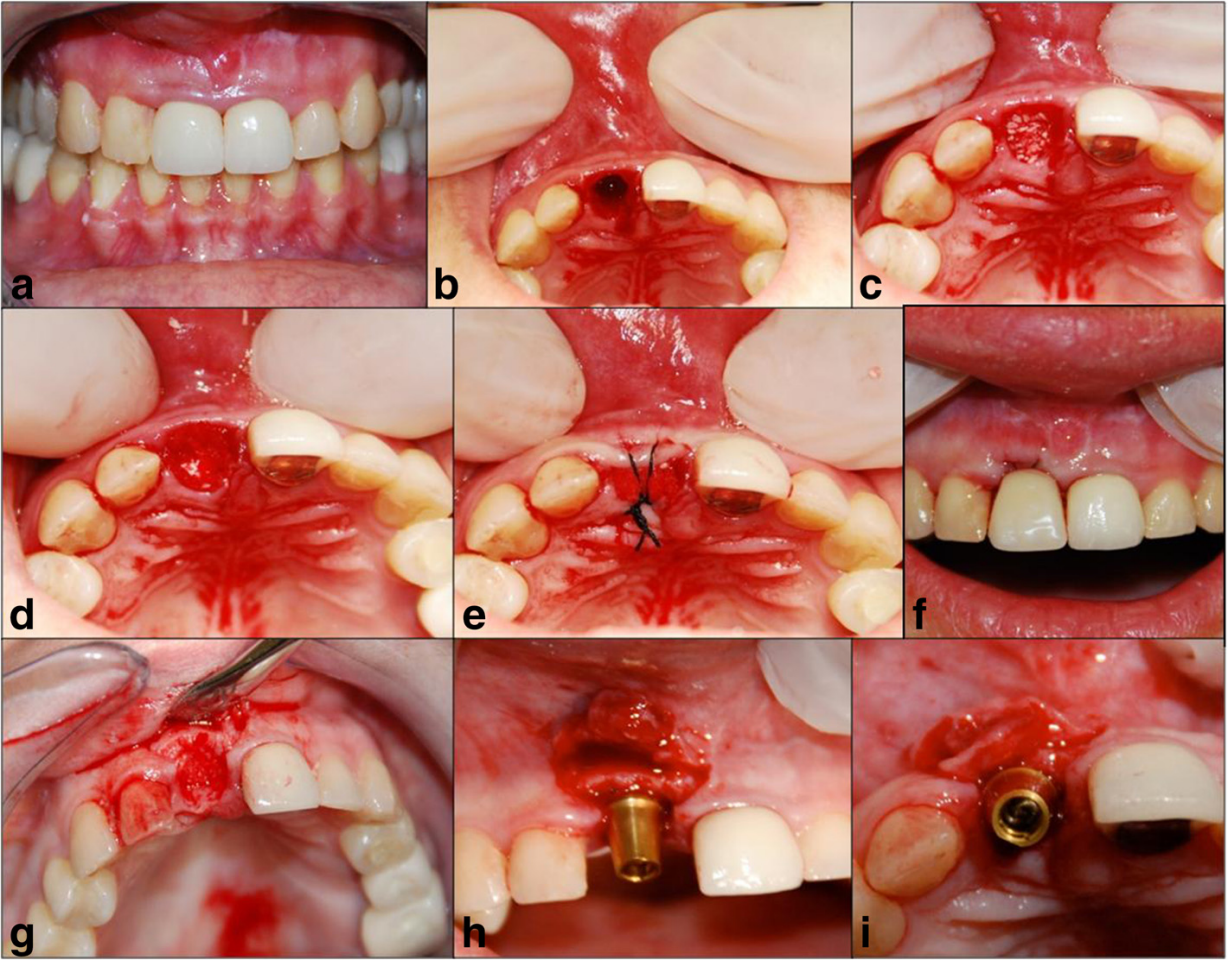
**a** Preoperative view failing root canal therapy on number 1.1, mobility 2+; (**b**) A-traumatic extraction following the surgical protocol bleeding in the socket was obtained with ½ round bur and copious irrigation; (**c**) Socket grafting using wetted particulate allograft material; (**d**) Extraction socket grafted with particulate allograft covered with a collagen dressing material Colla-Plug; (**e**) Cross mattress suture to stabilize the graft material; (**f**) Acrylic flipper used to protect the wound and for aesthetic purposes; (**g**) Collection of tissue biopsy after healing period; (**h**, **i**) Correct implant placement in the grafted socket after the tissue biopsy was collected from mid socket

All the patients were received an a-traumatic extraction, following the protocol described by Wang et al. [16]. The following tooth sites were considered as long as the remaining socket was intact: single-rooted (anterior teeth, posterior teeth or teeth with fused roots). The most common reasons for tooth extraction were: coronar fracture, profound decay, tooth mobility which do not damage the wall socket after extraction. Teeth with periapical lesions were excluded. In order to decrease the variability in the results only 5 wall extraction/wall defects were considered for this study. Two patients with a missing wall caused by infection or surgical trauma were removed from the study. Only areas with primary or secondary closure were included in the study. Each patient had a socket grafted with a particulate allograft material (MinerOss, Biohorizons, Canada) covered with a resorbable collagen wound dressing (CollaPlug, Zimmer Dent - for smaller extraction sites and MemLok, Biohorizons - for larger extraction sites), either in the maxilla or in the mandible (Fig. 1). Three patients with immediate complications after grafting such as loose membrane, loose bone graft material, etc. were excluded from the study.

All patients were pre-medicated with 800 mg Ibuprophen, 2000 mg Amoxicillin (or 600 mg Clindamycin in case of allergy to Amoxicillin) and 8 mg Dexomethasone 1 h before the extraction.

Postoperative instructions were given to the patients and included rinsing twice with warm salt water for the first 2 weeks before switching to with chlorhexadine gluconate 0.12 %, twice daily, for the next 2 weeks. Postoperative Ibuprophen 600 mg or Tylenol was recommended to control pain. Patients also received Dexomethasone 6 mg in day 1, 4 mg in day 2 and 2 mg in day 3 post-extraction. All patients were reappointed for suture removal 10–14 days post-extraction and grafting. 2–3 weeks postoperative all sockets showed uneventful healing with most of the surface of the soft tissue covered. The healing process was monitored periodically.

The patients were randomly divided in two equal groups (*n* = 15) using block randomization method: test group receiving postoperative treatment with the OsseoPulse phototherapy, delivery by operators, at an intensity of 20 mW/cm^2^ for 20 min per day for 21 consecutive days, and control group without postoperative laser treatment.

The assessment of bone formation was carried out in both groups at various time intervals after surgery by the means of a trephine, biopsy of tissue sampled at midpoint, followed by a histological analysis. All patients were scheduled for biopsies. The harvesting of the samples were possible in all patients in both groups. The biopsy in the control group were harvested at day 120, and in the test group were harvested at day 60. The biopsy time were determined radiologically.

The treatment performed was in the best interest of the patients. No biopsies were taken without immediate placement of a dental implant. If a site could be biopsied without compromising the long term success of the dental implant, the biopsy was carried out as described above. If the situation dictated otherwise (not proper healing time), the site was not biopsied until a later date. The welfare of the patient was the main criteria for the biopsy timing.

The harvested samples were immediately placed in 10 % formaldehyde fixative, decalcified in ethylene diaminetetracetic acid, dehydrated in increasing concentrations of ethanol, embedded in paraffin and cut sagittally. The sections were stained with Hematoxylin-Eosin and examined microscopically (Leitz DM - RBE Microscope, Leica Wetzlar Germany) at different magnifications (X6.3, X10, X25) by a trained, calibrated and blind to the groups evaluator.

## Results

From the 35 patients included in the study, five were drop-out, two due to missing wall caused by surgical trauma and three with immediate complications after grafting.

**Fig. 2:**
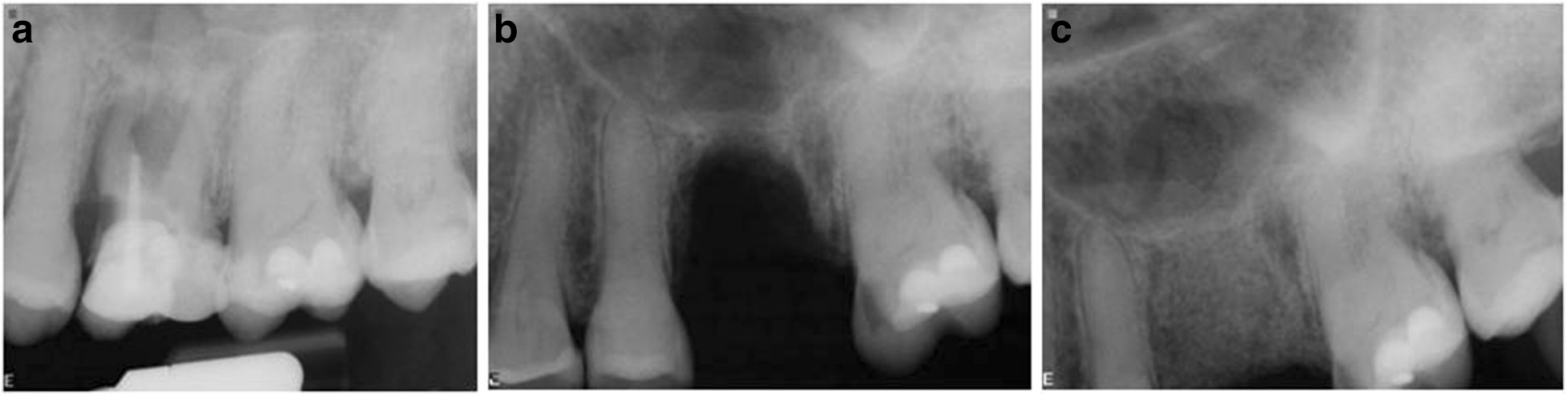
Radiographic evaluation. **a** at the extration date - preoperative view; (**b**) defect after the extraction; (**c**) grafted area with MinerOss and Mem-Lock membrane at 120 days post-operative

In the control group, not receiving LLLT biopsies were harvested after 120 days, and a complete turnover of the grafted material into woven bone was noticed on radiographic evaluation (Fig. 2).

**Fig. 3:**
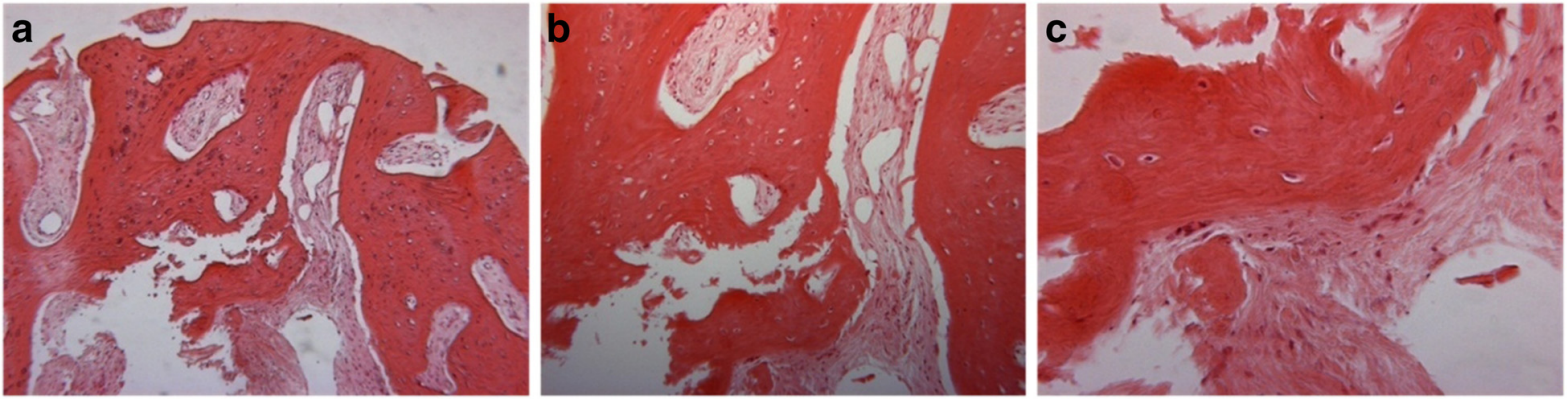
Biposy taken from a 43 years old subject in control group (non-treated with LLLT). **a** H& E stain, original magnification X6.3; (**b)** H& E stain, original magnification X10; (**c**) H& E stain, original magnification X25

The diagnosis of the biopsied site was interpreted to be vital woven bone. Histological examination revealed that the graft turnover - resorption and replacement by new bone -occurred rapidly with MinerOss cancelous and cortical bone chips. The new bone was not uniformly distributed throughout the core however most of it was histologically mature and the graft particles were integrated so that it was impossible to distinguish them from the new bone. High power photomicrograph showed that a lamellar pattern of mature bone had formed on the surface and surrounded the particles of MinerOss (Fig. 3a, b, c).

**Fig. 4:**
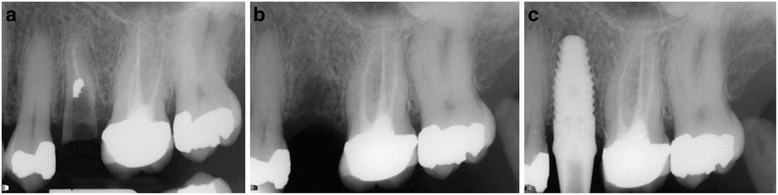
Radiographic imagines. **a** preoperative view, (**b**) 60 days post grafting; (**c**) final implant placement

Radiographic evaluation showed rapid bone regeneration in the test group (Fig. 4).

**Fig. 5:**
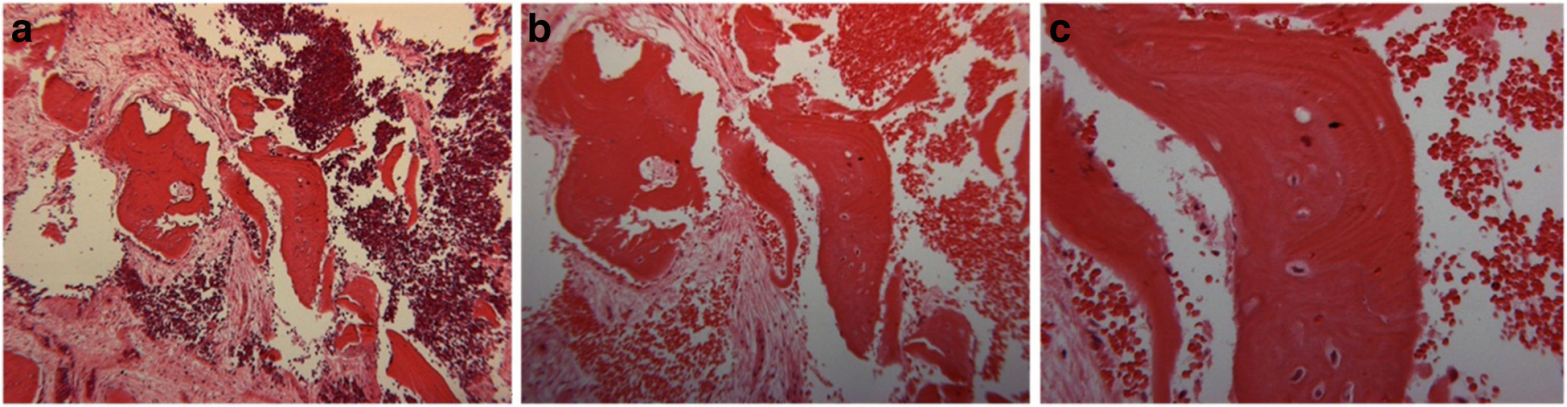
Biopsy taken from a 45 years old subject in study group (treated with LLLT). **a** H& E stain, original magnification X6.3; (**b**) H& E stain, original magnification X10; (**c**) H& E stain, original magnification X25

In the test group biopsies were harvested much sooner, that is 60 days after placement. The samples consisted of fragments of vascular fibrous connective tissue containing numerous bony trabeculae. The bony fragments were irregular in shape, some of which were interpreted to represent reactive bone formation showing numerous osteoblasts and osteocytes within the woven bone. Several fragments of vital laminar bone were also present. Occasional fragments of non vital laminar bone were present. No evidence of graft material was present. No significant differences in terms of vascularity of the regenerating bone between the groups was observed. The diagnosis of the biopsied site was interpreted to be reactive bone formation (Fig. 5a, b, c).

## Discussion

In our clinical study, the histological results of the sites treated with the LLLT for 21 days, harvested at 60 days after the grafting showed abundant new bone formation without any sign of inflammation. Osteoblasts and osteocytes were present in the woven bone. A vascular fibrous connective tissue was also present surrounding the numerous bony trabeculae. The presence of high amounts of collagen fibers in the test group may represent an early effect of the LLLT on bone repair [17]. Since the collagen fibers represent an important part of the extracellular matrix of the bone, the increase in amount can be an indicator of the positive effect of LLLT on bone regeneration. It can be considered that the large amount of the collagen can represent an increase in the bone formation after mineralization of the matrix.

Frozanfar [18] demonstrates that low level laser therapy stimulates human gingival fibroblast (HGF3-PI 53) proliferation and collagen type I gene expression *in vitro* which is in agreement with the results reported on the stimulatory effect of low laser irradiation on gingival fibroblast proliferation *in vitro* [19].

Graft matures into lamellar bone within a certain amount of time for healing depending on parameters such as: patient’s age, healing capacity, residual infection in the graft and the size of the defect. Generally, the healing period is considered 4–12 months. A previous study [20] suggested a healing period of over 4 months in order for the graft material (MinerOss, Biohorizons) to be resorbe and replace with a mature bone of the host. Enhancing and accelerating bone regeneration in the grafted extraction sockets would enable implant placement at a shorter time interval and therefore decrease the overall time of the treatment.

Tissue healing is a complex process that involves local and systemic organic activity, and fibroblasts are some of the cells directly involved in this mechanism. The action of lasers in healing is widely used therapeutic by inducing local and systemic regenerative, anti-inflammatory and analgesic effects [21, 22]. These effects have been demonstrated in vitro and in vivo particularly in studies that focus on the increase of local microcirculation, activity of the lymphatic system, proliferation of the epithelial cells and osteoblasts and increased collagen synthesis by osteoblasts [23, 24]. Pinheiro et al. [24] has suggested that although the benefits of laser in soft tissue healing have been demonstrated, the effects of laser on bone were controversial and the studies are conflicting.

LLLT has been applied in cell cultures and animal experiments on bone formation and have shown a positive effect on osteoblast proliferation and differentiation [25, 26]. A vitro study, performed by Stein et al. [5], showed that He-Ne Laser irradiation promotes proliferation and maturation of human osteoblasts. A number of studies also show a positive influence of the laser irradiation on wound healing [27] and collagen synthesis [28]. In addition, LLLT has been shown to moderate inflammation, stimulate HeLa cells proliferation [29] and angiogenesis [30].

A number of animal studies have shown the positive effect of the LLLT on bone repair and regeneration. Pinheiro et al. [17] assessed the effect of LLLT (wave length 830 nm) on repair of standardized bone defects on the femur of Wistar Albinus rats which were grafted with inorganic bovine bone Gen-ox. The results showed evidence of a more advanced repair in the irradiated group when compared to the non-irradiate group. The repair of the irradiated group was characterized by both increased bone formation and amount of collagen fibers around the graft within 15 days post-surgery. As the collagen is an important part of the extracellular matrix of bone the increased amounts of collagen in some specimens indicates a positive effect of the LLLT, even though the amount of new bone was the same in control and treated groups. The author concluded that LLLT had a positive effect on the repair of bone defects implanted with inorganic bovine bone.

The first human study was done by Brawn et al. [29] when he studied the effect of a red and near infrared (NIR) laser phototherapy on bone regeneration. Brawn, in these case report bilateral extraction sites were grafted with the synthetic hydroxyapatite (HA) particulate - OsteografLD300 (Dentsply Friadent CeraMed LAkeWOOD CO), one phototherapy treated and one untreated. The histological evaluation of the two sites showed an increased bone formation and faster particle resorption associated with the phototherapy treated site compared to the non-treated site. In a different clinical case study, Brawn et al. [30] studied the effect of a LED phototherapy on a sinus grafted with a particulate bovine bone material xenograft. A course of 20 mW/cm2 620 nm Light Emitting Diode (LED) phototherapy was performed for a period of 10 min two times per day for 2 weeks. After 4 weeks a biopsy was analysed histologically and it demonstrated a robust healing in response to the LED phototherapy.

Our results are in agreement with those of others authors [31], but further research need to be performed in order to identify the exact mechanisms of LLLT action on bone regeneration.

Limitation of the study are that we did not perfome histomorphometric analyses yet, the present study beeing a preliminary one, and the reduced number of patients. Further studies are necessary to sustain our results.

## Conclusions

LLLT has the ability to reduce healing time after grafting in the extraction sockets. Histological evidence suggests that in about 60 days there is new bone formation in the test group sockets compared to a minimum of 120 days in the control group. The LLLT has a positive biomodulatory effect on bone repair grafted with particulate allograft.

For the future, we propose to include a control group who will receive only LLLT with no socket grafting, since LLLT enhances “de novo” bone healing [29].

LLLT can be considered are useful method for reducing the oberall treatment time between extraction-implant placement. Although the patients need to visit the clinic for 21 consecutive days after surgery, with additional treatment costs, they consider that the benefit of LLLT treatment are higher in comparison with their efforts. Our histological results sustain the efficiency of LLLT for this purpose.

However, further studies are necessary to demonstrate the exact mechanism through which LLLT stimulates new bone formation.

## Abbreviations

LLLT: Low level laser treatment
NIR: Near-infrared laser phototherapy
HA: Hydroxyapatite
LED: Light emitting diode
