# Erratum to: Bone healing after low-level laser application in extraction sockets grafted with allograft material and covered with a resorbable collagen dressing: a pilot histological evaluation

**DOI:** 10.1186/s12903-016-0173-4

**Published:** 2016-02-09

**Authors:** Adriana Monea, Gabriela Beresescu, Sorin Boeriu, Mezei Tibor, Sorin Popsor, Dragos Mihai Antonescu

**Affiliations:** 1 Department of Odontology and Oral Pathology, Faculty of Dental Medicine, University of Medicine and Pharmacy Tirgu-Mures, Tirgu Mureș, Romania; 2 Department of Tooth Morphology and Dental Materials, Faculty of Dental Medicine, University of Medicine and Pharmacy Tirgu-Mures, Tirgu Mureș, Romania; 3 Department of Morphopatology, Faculty of Medicine, University of Medicine and Pharmacy Tirgu-Mures, Tirgu Mureș, Romania; 4 Department of Prosthetics and Oral Rehabilitation, Faculty of Dental Medicine, University of Medicine and Pharmacy Tirgu-Mures, Tirgu Mureș, Romania; 5 grid.67105.350000000121643847Resident Graduate Periodontal Program, School of Dental Medicine, CaseWestern Reserve University, Cleveland, Ohio USA

After publication of this study [1], we realize that Dr. Sorin Boeriu was not listed among the authors. Please see the corrected author list below:

Adriana Monea†, Gabriela Beresescu *†, Sorin Boeriu, Mezei Tibor, Sorin Popsor and Dragos Mihai Antonescu
